# In vitro assessment of histamine and lactate production by a multi-strain synbiotic

**DOI:** 10.1007/s13197-021-05327-7

**Published:** 2021-12-02

**Authors:** Gerrit Stuivenberg, Brendan Daisley, Polycronis Akouris, Gregor Reid

**Affiliations:** 1 Centre for Human Microbiome and Probiotic Research, Lawson Health Research Institute, Room F3-106, 268 Grosvenor Street, PO BOX 5777 STN B, London, ON N6A 4V2 Canada; 2 Department of Microbiology and Immunology, Western University, London, ON N6A 3K7 Canada; 3 Department of Biochemistry, Western University, London, ON N6A 3K7 Canada; 4 Department of Surgery, Western University, London, ON N6A 3K7 Canada

**Keywords:** Probiotic, Synbiotic, Histamine, Lactate, Food Safety

## Abstract

Recent studies suggest histamine and d-lactate may negatively impact host health. As excess histamine is deleterious to the host, the identification of bacterial producers has contributed to concerns over the consumption of probiotics or live microorganisms in fermented food items. Some probiotic products have been suspected of inducing d-lactic-acidosis; an illness associated with neurocognitive symptoms such as ataxia. The goals of the present study were to test the in vitro production of histamine and d-lactate by a 24-strain daily synbiotic and to outline methods that others can use to test for their production. Using enzymatic based assays, no significant production of histamine was observed compared to controls (*P* > 0.05), while d-lactate production was comparable to a commercially available probiotic with no associated health risk. These assays provide a means to add to the safety profile of synbiotic and probiotic products.

## Introduction

**Fig. 1:**
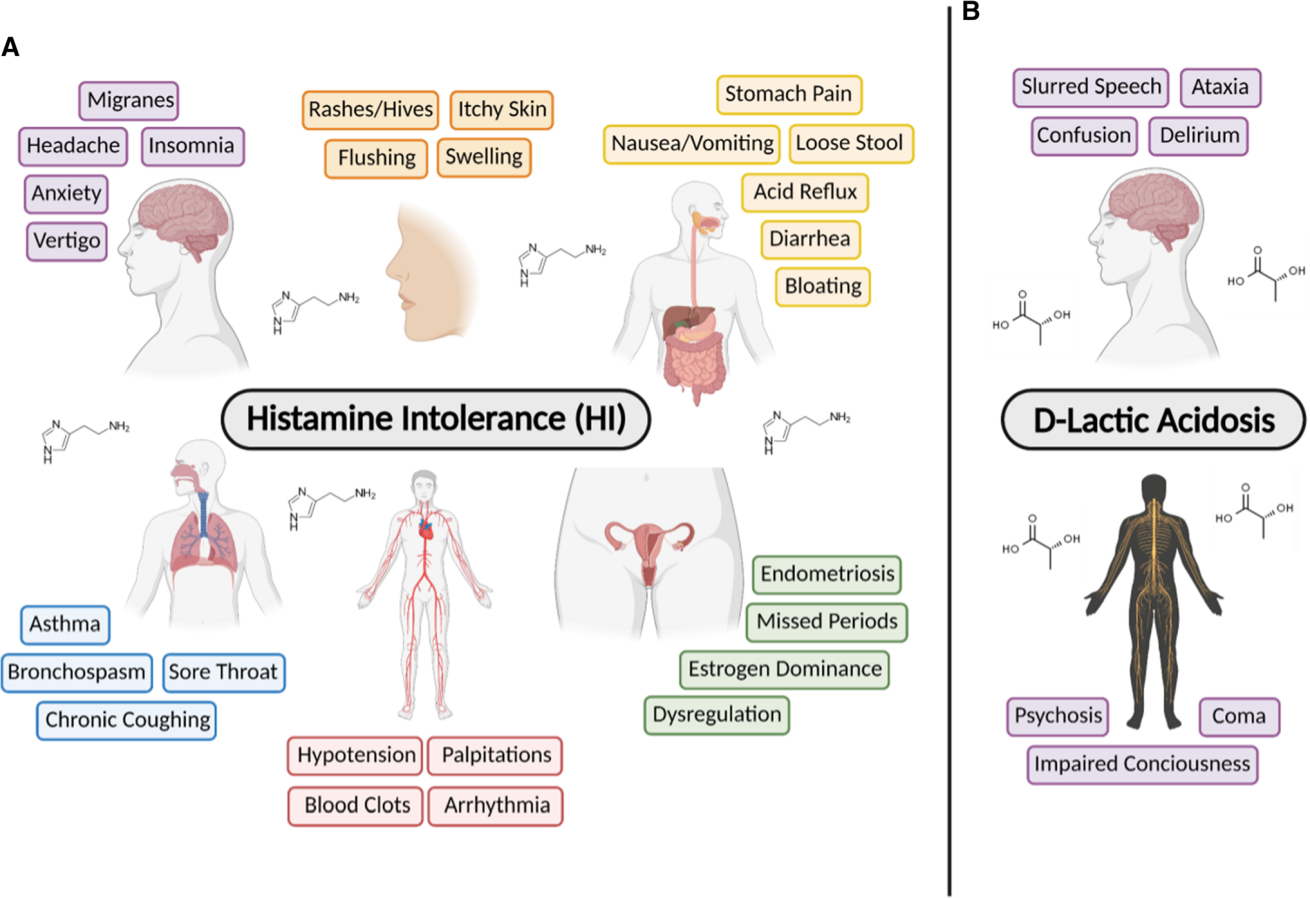
The microbial biomolecules (**a**) histamine and **b** d-lactate can be deleterious to multiple organ systems

Probiotics are defined as ‘live microorganisms that, when administered in adequate amounts, confer a health benefit on the host’ (Hill et al. 2014). Since these organisms are alive at the time of administration, there is the potential to cause infection or to produce compounds that may be harmful to the host (Sanders et al. 2010). A deficiency in regulatory monitoring has resulted in the inappropriate labelling of some commercial probiotics, including species and strain designation (de Simone 2019). This is disconcerting because the health promoting effects of probiotics are strain-specific (Hill et al. 2014) and depend on the disease state of the recipient (McFarland et al 2018). Often, companies combine strains in an ad hoc manner without considering that some might interfere with each other and alter the effect they have on host physiology. Well-studied compounds like histamine and D-lactate, produced by various probiotic microorganisms (de Simone 2019; Maintz and Novak 2007; Morrow et al. 1991), have the potential to negatively impact host health (Fig. 1).

Histamine intolerance (HI) can develop following the prolonged accumulation of histamine in the body. Symptoms of HI range from headaches and flushing, to life-threatening conditions such as hypotension, bronchospasm, and shock (Morrow et al. 1991; Sattler et al. 1989; Sattler and Lorenz 1990). Gastrointestinal bleeding can enhance the accumulation of histamine in the body as can ingesting histamine and histidine rich foods, and gut colonization by histamine-producing bacteria (Maintz and Novak 2007). Although orally supplemented probiotics do not colonize the gastrointestinal tract, their temporary presence might influence histamine production by the host (Liu et al. 2018). Thus, it is imperative to verify that histamine production by probiotic strains is insufficient to cause illness within the context of the target disease, prior to clinical studies.

In humans, lactate is a common by-product of anaerobic metabolism and it exists as two isoforms—l-lactate and D-lactate (Petersen 2005). High titres of d-lactate (> 3 mM/L) in the blood can cause d-lactic acidosis, a rare condition that induces slurred speech, ataxia, and sometimes coma by impacting the central nervous system (Petersen 2005; Uribarri et al. 1998). Although the production of D-lactate by human cells is negligible, some bacteria in the gut are capable of generating this isoform at biologically-relevant levels via fermentative processes (Petersen 2005). Lactate-producing bacteria make either one or both isoforms and are deemed homofermentative or heterofermentative, respectively. Hence, the ratio of bacteria producing each isoform will impact absolute and relative concentrations of d-/l-lactate in the body (Hove and Mortensen 1995). Rao et al (2018) claimed that probiotic supplementation in patients with small intestinal bacterial overgrowth (SIBO) is responsible for d-lactic acidosis and is associated with neurological deficits, gas, and bloating. While these conclusions are poorly supported by their data, attention gained through the publicization of these and other radical claims have raised concern towards probiotics that can produce lactate (Doron and Syndman 2015; Quigley et al 2018; Shi et al. 2016).

The genus *Lactobacillus* is one of the largest contributors to the collection of strains used as probiotics (Shi et al. 2016). Denoting the vast genomic and functional differences within this supergroup of bacteria, *Lactobacillus* has recently been reclassified into 25 separate genera while retaining specific epithets of their original binomial nomenclature (e.g., *Lactobacillus rhamnosus* is now *Lacticaseibacillus rhamnosus*) (Zheng et al. 2020). Objectively, certain species of this supergroup are capable of generating histamine and D-lactate via the conversion of histidine using bacterial histidine decarboxylase and anaerobic respiration, respectively (Pot et al. 2014; Wüthrich et al. 2017). Accordingly, this could yield risks associated with the consumption of these microbes. However, the extent of this risk must be judged at the level of individual supplements to avoid blanket statements that condemn all probiotics. Without considering the variation that exists between their contents, function, and metabolic output that results from the inclusion/exclusion of certain strains, the unique effect of a probiotic on the host is ignored. In other words, a probiotic could potentially be beneficial or detrimental to the health status of the host depending on the disease state. Unfortunately, suggesting that probiotics in general may be harmful based on poorly drawn conclusions from a diseased population, like those with SIBO, may prevent individuals from considering clinically proven therapies. As such, there is a dire need for rigorous protocols that assess the safety of probiotic formulations by identifying both the metabolic profile (i.e. potentially harmful metabolites) of these products and the at-risk populations that should avoid them.

The goals of the present study were to examine a relatively new multi-strain synbiotic for the ability to produce d-lactate and histamine as an addition to its safety profile and, to outline fast and simple methods that can be used to test commercially available strains and multi-strain formulations in the future.

## Material and methods

### Culture condition for histamine analysis

Individual synbiotic capsules (Seed Health, California) that contain 24 strains of probiotic bacteria (Table 1) were opened and the contents were aseptically added to 50 mL conical tubes containing 45 mL of De Man, Rogosa and Sharpe (MRS) liquid broth media (Sigma Aldrich). All of the included strains were known to grow in this medium. The reason for testing all the strains together was to simulate what would occur upon human consumption. The tubes were vortexed for 30 s to ensure the contents of the capsules were dissolved and equally dispersed throughout the media. Samples were then incubated at 37 °C under stationary conditions in an aerobic or anaerobic environment (BD GasPak™; catalog #: 260,678) for 96 h to assess histamine production under varying levels of oxygen.

**Table 1:** Composition of screened probiotics

Product	Strain	Colony Forming Units
Renew – Ultimate Flora Vaginal Support 50 Billion	*Bifidobacterium infantis* Bi-26 (ATCC SD-6720) *Bifidobacterium lactis* Bi-04 (ATCC SD-5219) *Bifidobacterium lactis* BB-12 (DSM 15954) *Lacticaseibacillus rhamnosus* GG (ATCC 53103) *Lacticaseibacillus rhamnosus* GR-1 (ATCC 55826) *Lacticaseibacillus casei* Lc-11 (ATCC SD5213) *Lacticaseibacillus paracasei* Lpc-37 (ATCC SD5275) *Lactiplantibacillus plantarum* Lp-115 (ATCC SD5209 *Lactobacillus acidophilus* La-14 (ATCC SD5212) *Lactococcus lactis* Ll-23 (ATCC SD5584) *Levilactobacillus brevis* Lbr-35 (ATCC SD5214) *Limosilactobacillus reuteri* RC-14 (ATCC 55845)	Total probiotic viable count, 50 billion with no strain under 1 billion
Seed synbiotic Contains: Microbiota-accessible prebiotics 190 mg: polyphenol pomegranate (whole fruit and skin with 30% punicalagins), organic pine bark flavonoids (50% oligomeric proanthocyanidins), organic chaga mushroom polysaccharides	*Bifidobacterium adolescenti*s SD-BA5-IT (DSM 18352) *Bifidobacterium breve* SD-BR3-IT (DSM 16604) *Bifidobacterium breve* HRVD521-US* *Bifidobacterium infantis* SD-M63-JP* *Bifidobacterium lactis* HRVD524-US* *Bifidobacterium longum* SD-BB536-JP* *Bifidobacterium lactis* SD-BS5-IT (LMG P-21384) *Bifidobacterium lactis* SD150-BE* *Bifidobacterium lactis* SD-CECT8145-SP (CECT 8145) *Bifidobacterium lactis* SD-MB2409-IT (DSM 23733) *Bifidobacterium longum* HRVD90b-US* *Bifidobacterium longum* SD-CECT7347-SP (CECT 7347) *Lacticaseibacillus casei* HRVD300-US *Lacticaseibacillus casei* SD-CECT9104-SP (CECT 9104) *Lacticaseibacillus rhamnosus* HRVD113-US* *Lacticaseibacillus rhamnosus* SD-GG-BE (ATCC 53,03) *Lacticaseibacillus rhamnosus* SD-LR6-IT (DSM 21980) *Lactiplantibacillus plantarum* 2830 (ECGC 13110402) *Lactiplantibacillus plantarum* SD-LP1-IT (LMG P-21021) *Ligilactobacillus salivarius* SD-LS1-IT (DSM 22775) *Limosilactobacillus fermentum* SD-LF8-IT (DSM 18297) *Limosilactobacillus reuteri* RD830-FR* *Limosilactobacillus reuteri* SD-LRE2-IT (DSM 23878)	Total probiotic viable count, 53.6 billion with no strain under 1 billion

*Have been deposited to the ATCC and are awaiting deposition number

*Limosilactobacillus reuteri* ATCC 23272, used as a positive control, was streak plated from the frozen stock culture onto MRS agar and was incubated anaerobically at 37 °C overnight. A single colony was selected and inoculated for 12 h at 37 °C in MRS broth under anaerobic conditions. Subsequently, the overnight cultures were sub-cultured (1:225 dilution) into fresh MRS broth media. Cultures were then incubated anaerobically at 37 °C for 96 h prior to histamine analysis.

### Histamine quantification

A competitive enzyme-linked immunosorbent assay (ELISA) was used to quantify the concentration (ng/mL) of histamine in each sample. Aliquots of 1 mL from each sample were centrifuged at 1000 *g* for 20 min at 4 °C. Subsequently, the supernatant was used to quantify histamine following the manufacturers’ instructions (Histamine ELISA Kit; E-EL-0032; Elabscience).

### Culture conditions for D-/L-lactate analysis

The Seed synbiotic was tested for D-lactate production. Individual synbiotic capsules were opened and the contents were aseptically transferred to 50 mL conical tubes containing 45 mL of MRS broth media. The tubes were vortexed for 30 s to homogenize the contents of the capsules and ensure equal distribution throughout the media. Samples were then incubated anaerobically under stationary conditions at 37 °C for 24 h. Subsequently, bacterial cells were centrifuged (5000 *g* for 10 min) and washed twice with 1× phosphate-buffered saline (8 g NaCl, 0.2 g KCl, 1.44 g Na_2_HPO_4_, and 0.24 g KH_2_PO_4_ dissolved in 1 L H_2_O; pH 7.4; PBS) and once with Krebs–Ringer Buffer (8.47 g NaCl, 683.89 mg NaH_2_PO_4_, 362.32 mg KCl, 59.93 mg CaCl_2_, 149.85 mg MgSO_4_, and 990.86 mg glucose dissolved in 1 L H_2_O; pH 7.35). After being washed, the cells were transferred to a 50 mL conical tube containing 45 mL of Krebs–Ringer Buffer, which facilitates metabolic activity of bacterial cells (Rodbell 1964; Krebs and Henseleit 1932), and were incubated aerobically or anaerobically at 37 °C. l-/d-lactate production was subsequently measured in the samples after 1 and 24 h of incubation.

A nine-strain product (Renew Ultimate Flora; Table 1) was used as a multi-strain control to highlight the production of lactate by these products; it was prepared as described above. Single strain controls, *Lacticaseibacillus rhamnosus* GG (ATCC 53,103) and *Lactobacillus gasseri* ATCC 33,323 were streak plated from the frozen stock cultures onto MRS agar and were incubated anaerobically at 37 °C overnight under stationary conditions. A single colony was selected and inoculated for 12 h at 37 °C in MRS liquid media. Subsequently, the overnight culture was sub-cultured (1:225 dilution) into fresh MRS broth and was incubated anaerobically for 24 h at 37 °C. The bacterial cells were centrifuged (5000* g* for 10 min) and washed twice with 1 × PBS and once with Krebs–Ringer Buffer. After being washed, the cells were transferred to a 50 mL conical tube containing 45 mL of Krebs–Ringer Buffer and were incubated at 37 °C under anaerobic or aerobic conditions. d-/l-lactate production was then measured in samples after 1 and 24 h of incubation.

### Quantification of d-/l-lactate

A standard enzymatic assay, based on the conversion of lactate to pyruvate in the presence of NAD and lactate dehydrogenase (LDH), was used to quantify the concentration of d-/l-lactate in the samples (Vanderlinde 1985). After 1 or 24 h of incubation, the bacterial cell cultures were centrifuged at 5000* g* for 10 min at room temperature. Subsequently, 20 μL of supernatant aliquots were collected and transferred to a flat-bottom 96 well assay plate with each well containing 250 μL buffer solution containing 0.4 M glycine (Invitrogen; catalog #: 15,527–013), 0.5 M hydrazine (Sigma; catalog #: 216,046), 25 μL NAD (17 mg/mL) (Roche; catalog #: 10,127,981,001), and 2.5 μL of either D-LDH (Sigma; catalog #: L3888) or L-LDH (Roche; catalog #: 10,127,230,001). After the addition of the culture supernatants, the plate was incubated for 1 h at 25 °C. Following incubation, optical density was measured at 340 nm (OD340) using a BioTek PowerWave HT microplate reader (BioSPX). Values were standardized to total protein in the sample.

### Determination of protein concentration in culture supernatants

Total protein content in the supernatants of the tested cultures was determined using a Pierce BCA Protein Assay Kit (Thermo Scientific) following the manufacturer’s instructions. Briefly, 25 μL of each sample (aliquoted from the same supernatant extracted during previous lactate quantification steps) was added to 200 μL of the working reagent, mixed thoroughly, and then incubated under stationary conditions in the dark at 37 °C for 30 min. Subsequently, colorimetric detection and quantitation of total protein were determined by measuring optical density at 562 nm using a BioTek PowerWave HT microplate reader (BioSPX).

### Statistical analysis

All statistical comparisons were performed using GraphPad Prism 9.0 software. Data values were tested for normality using the Shapiro-Wilks test or D’Agostino and Pearson normality test. Non-parametric data were statistically compared with an unpaired, one-way Kruskal–Wallis test, complemented with Dunn’s multiple comparison test. Normally distributed data were compared with an unpaired, one-way analysis of variance (ANOVA), complemented with Tukey’s multiple-comparison test.

## Results and discussion

This is the first study to advocate for and to outline the necessary methods to assess the production of potentially harmful microbial metabolites by probiotic products. This study showed that histamine production by Seed synbiotic was undetectable despite containing a *L. reuteri* strain, which at a species level has been reported to produce histamine (Hemarajata et al. 2013). Both isoforms of lactate were produced by Seed synbiotic, but the concentrations were not different from another probiotic, Renew, that despite widespread use has not been shown to cause biomolecule associated complications.

### Histamine production

**Fig. 2:**
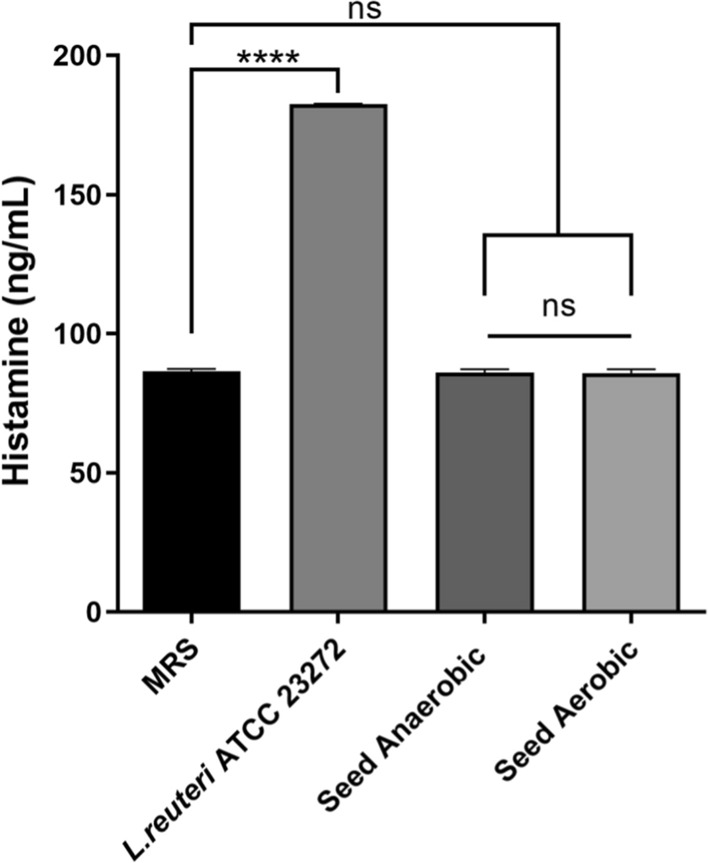
In vitro production of histamine by *L. reuteri* ATCC 23,272 and Seed synbiotic grown aerobically and anaerobically in MRS. Statistical significance was determined by comparing each test group to the uninoculated control. Significance is indicated by asterisk (**** = *p* < 0.0001) or by lettering (ns = *p* > 0.05)

At normal serum concentrations, histamine is an important mediator of multiple biological processes including immunomodulation in humans (Maintz and Novak 2007). Recently, there have been reported cases of histamine intolerance developing in individuals that consumed a probiotic product containing histamine producing microbes (Liu et al., 2018). This is counter-intuitive since the aim of probiotics is to improve host health. The mean histamine content of Seed synbiotic when cultured anaerobically and aerobically was 85.99 ± 1.23 and 85.76 ± 1.42 ng/mL, respectively (Fig. 2). This was similar to the negative control, uninoculated MRS media, which contained 86.44 ± 0.92 ng/mL of histamine (Fig. 2). No significant difference in histamine content was observed after comparing Seed synbiotic samples, cultured both aerobically and anaerobically, to the uninoculated control (ANOVA, *P* > 0.05; Fig. 2). *L. reuteri ATCC* 23,272, previously shown to produce histamine (Mu et al. 2018), demonstrated significantly higher histamine content in its culture supernatant compared to all of the synbiotic groups and the uninoculated control (ANOVA, *P* < 0.0001; Fig. 2).

The histamine observed in the synbiotic samples was accounted for by the background levels present in the MRS broth media used for culturing (Fig. 2). This medium contains beef extract, a product with defined histamine content (Man et al. 1960; Bermudo et al. 2004). Given there was no observable difference in histamine concentration between the vehicle control and cultures of Seed synbiotic, the amount of histamine produced by the probiotic strains included in the formulation is negligible. Thus, the risk of healthy adults developing HI following consumption of this product is insignificant.

### Lactate production

**Fig. 3:**
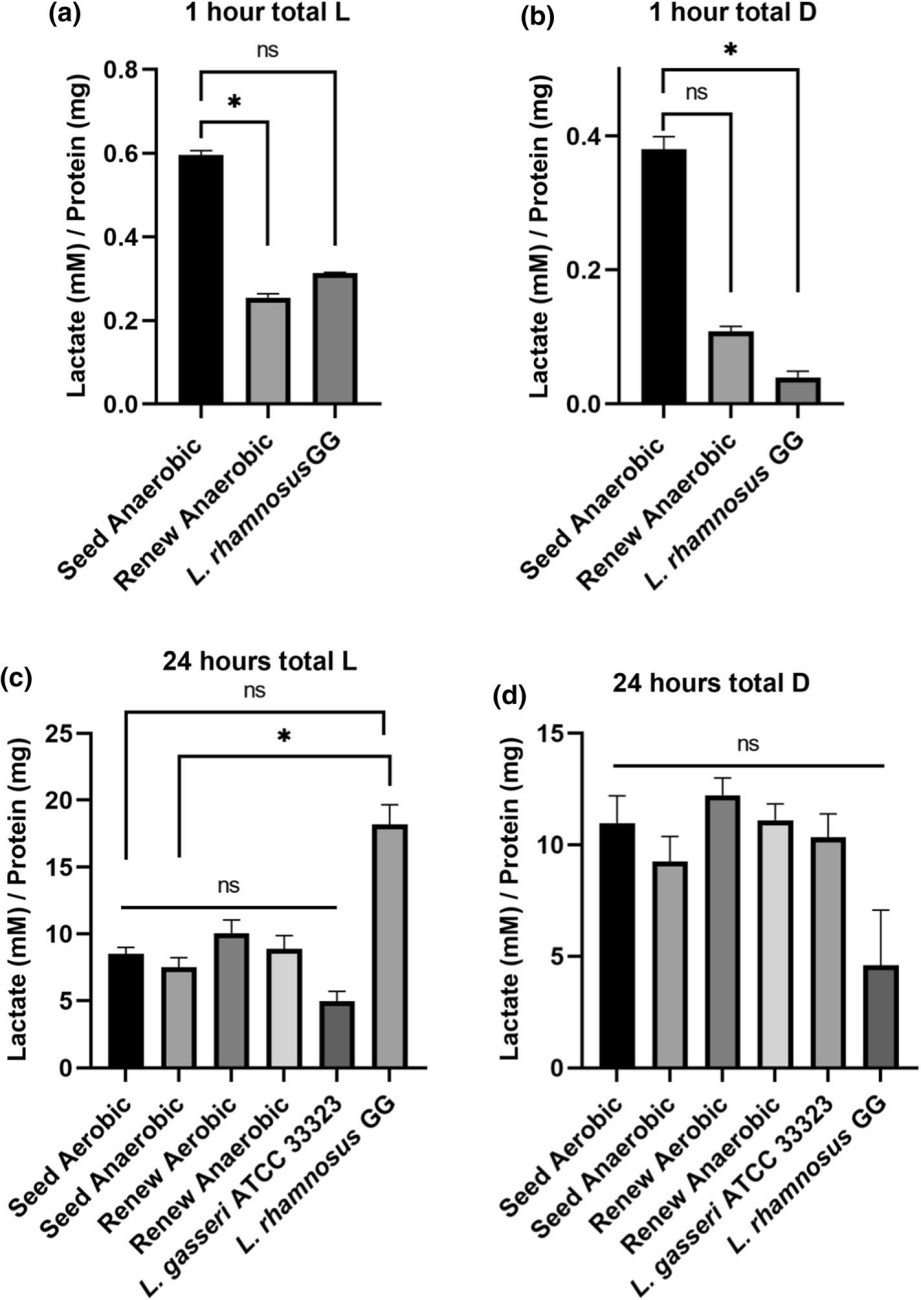
In vitro production of **a** l-lactate and **b** D-lactate after 1 h and **c** l-lactate and **d** d-lactate after 24 h by two probiotic products and *Lactobacillus* strains in a relevant buffer solution. Statistical significance was determined by comparing each Seed synbiotic test group to a relevant probiotic mixture (Renew) and single strain controls. Significance is indicated by asterisk (* = *p* < 0.05) or by lettering (ns = not significant, *p* > 0.05)

**Fig. 4:**
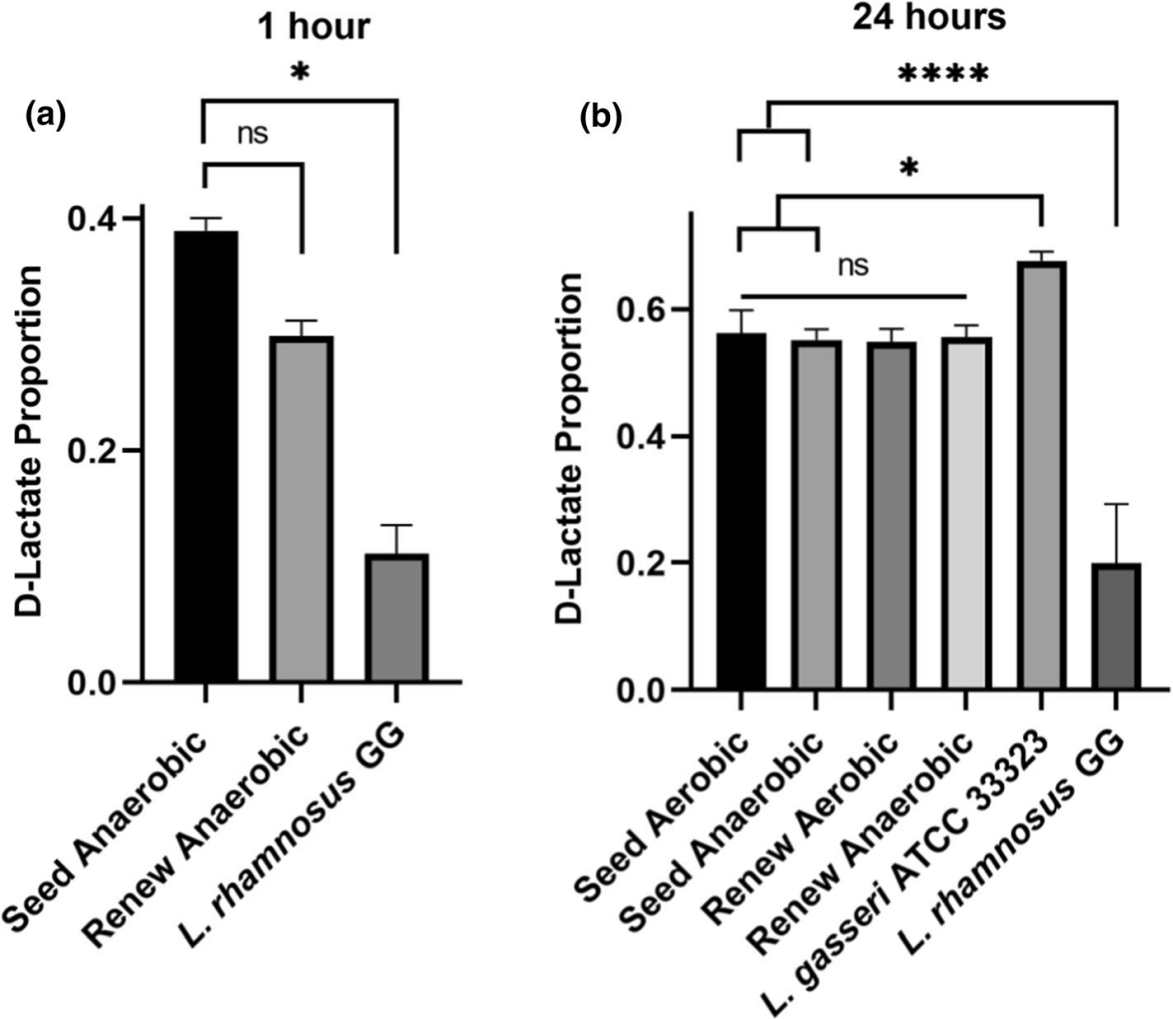
The proportion of D-lactate from the total lactate produced in vitro after **a** 1 h and **b** 24 h by two probiotic products and *Lactobacillus* strains in a relevant buffer solution. Statistical significance was determined by comparing each Seed synbiotic test group to a relevant probiotic mixture (Renew) and single strain controls. Significance is indicated by asterisk (* = *p* < 0.05 and **** = *p* < 0.0001) or by lettering (ns = not significant, *p* > 0.05)

A report by Rao et al. (2018) condemned probiotic supplements as a general risk factor for the development of D-lactic acidosis-induced neurocognitive deficits. However, the conclusions are largely unsupported by the dataset; duodenal screening of symptomatic patients for D-lactate producing bacteria showed that common probiotic organisms such as *Lactobacillus* and *Bifidobacterium* spp. were rarely present, if at all. Furthermore, even though approximately 3.9 million people consume probiotics daily, in the US alone, only ~ 5 cases of probiotic associated D-lactic acidosis have been reported to date (Doron and Snydman 2015). Despite the rarity of the condition and a lack of convincing evidence, these claims have caused concern amongst consumers (Doron and Snydman 2015; Shi et al. 2016; Quigley et al. 2018). Accordingly, the production of both lactate isoforms by Seed synbiotic was investigated. The amount of lactate produced after 1 and 24 h incubation periods was determined for each sample (Fig. 3) and the relative ratio of the l-/d-forms was calculated (Fig. 4).

At the 1 h timepoint, every product and strain tested produced more L-lactate than D-lactate. The supernatants from the Seed synbiotic cultures contained the greatest quantity of both the l-/d-lactate isoforms with a mean of 0.59 ± 0.01 and 0.38 ± 0.01 mM, respectively (Fig. 3a, b). Renew probiotic yielded 0.25 ± 0.01 mM of l-lactate and 0.10 ± 0.01 mM of D-lactate (Fig. 3a, b). Despite Seed producing more lactate than Renew, their respective L:D ratios of total lactate produced were not significantly different from each other (Kruskal–Wallis, *P* > 0.05; Fig. 4a).

After 24 h, every product and strain tested favoured the production of d-lactate over the L-isoform, except *L. rhamnosus* GG (ATCC 53,103). The supernatants of the Renew probiotic cultures contained more D-lactate under both aerobic (12.21 ± 0.78 mM) and anaerobic (11.09 mM ± 0.74) conditions (Fig. 3c and d). However, this was not statistically significant from any of the other tested groups (Kruskal–Wallis, *P* > 0.05; Fig. 3d). The total L:D ratio of both Seed and Renew were highly similar after 24 h (Fig. 4b). This suggests that the prebiotics present in Seed Synbiotic did not influence lactate metabolism. *L. rhamnosus* GG (ATCC 53103), a strain that predominantly produces the L-isoform of lactate (Manome et al. 1998), consistently produced greater amounts of L-lactate at both 1 h and 24 h (Fig. 3) and demonstrated similar L:D ratios at both time points (Kruskal–Wallis, *P* < 0.05, Fig. 4).

While our data shows that the Seed synbiotic mixture and the Renew product produce D-lactate (Fig. 3), it is in nearly equimolar ratio with L-lactate production (Fig. 4a and b). Both of the lactate isoforms are absorbed by the small intestinal and colonic epithelial cells in mammals via the monocarboxylate transporter 1 (MCT-1) (Ding and Xu 2003). Importantly, MCT-1 is saturable with both l- and d-lactate exhibiting mutual inhibitory effects on each other, whereas the uptake coefficient of L-lactate is twice that of d-lactate (Tamai et al. 1995). Accordingly, it is expected that the presence of l-lactate will effectively reduce d-lactate uptake by the body through competitive inhibition of MCT-1 and that equimolar production of D-/L-lactate by probiotics should not be a real concern to healthy consumers.

The sparsity of reports linking microbial lactate to illnesses might be further explained by the natural processing of the compound in the body. Lactate is seldom the final fermentation product of mixed anaerobic communities like those defined in the human gut and it may be converted to molecules that are beneficial to host health, like butyrate (Duncan et al. 2004) This indicates that lactate produced by Seed synbiotic is likely to be managed by members of the gut microbiota or excreted in the feces. Corroborating this, no cases of D-lactic acidosis have been reported for the Seed and Renew products nor any other commercially available, clinically documented, products containing multiple strains of lactate-producing bacteria. Hence, the risk of the Seed product causing lactate associated illnesses in healthy adults is negligible.

## Conclusion

In summary, these findings add to the safety profile of a 24 strain synbiotic and provide valuable information to interested consumers. This study outlines assessment methods for histamine and d-lactate production in commercial products. Although these methods cannot perfectly replicate how histamine and d-lactate are produced in the human gut, they provide adequate insight to the relative risk of the products in question. Therefore, these tests can identify probiotics strains or products that produce concerning amounts of these compounds and will be useful to guide further safety analysis and supplementation protocols. Given that lactobacilli may increase the production of these compounds, such tests should be considered mandatory as part of a safety assessment for any probiotic products containing these species, thereby offering transparency to the consumer. The creation of products termed probiotic requires improved communications between the supplier of the strains and the retailer, to carefully document the expectation of the strains. Thereafter, clear communication on expected mechanisms of action and safety profile should be relayed to the consumer. Consumers should take the time to examine products and their scientific validity and be wary of headlines that generalize the whole field or make unsubstantiated claims.

## Data Availability

The datasets generated and/or analysed during the current study is not publicly available due to privacy concerns but are available from the corresponding author upon reasonable request.

## Abbreviations

HI: Histamine intolerance
SIBO: Small intestinal bacterial overgrowth
MCT-1: Monocarboxylate transporter
